# Effect of salt stress on the photosynthetic characteristics and endogenous hormones, and: A comprehensive evaluation of salt tolerance in *Reaumuria soongorica* seedlings

**DOI:** 10.1080/15592324.2022.2031782

**Published:** 2022-02-22

**Authors:** Shipeng Yan, Peifang Chong, Ming Zhao

**Affiliations:** a College of Forestry, Gansu Agricultural University, Lanzhou, China; b Gansu Province Academy of Qilian Water Resource Conservation Forests Research Institute, Zhangye, China

**Keywords:** *Reaumuria soongorica*, salt stress, growth and development, photosynthetic characteristics, endogenous hormones

## Abstract

Salinity is a major limiting factor in desert ecosystems, where *Reaumuria soongarica* is a dominant species. It is crucial to study the growth and physiological response mechanisms of *R. soongorica* under salt stress for the protection and restoration of the desert ecosystems. However, the effects of salt concentration and stress duration on endogenous hormonal content and photosynthetic efficiency and salt injury index of *R. soongorica* leaves have not been reported. Currently, there is no systematic evaluation system to determine physiological adaptation strategies of *R. soongorica* seedlings in response to salt stress. In this study, simulation experiments were performed with NaCl solution mixed with soil. Enzyme-linked immunosorbent assay and LI-6800 portable photosynthesis analyzer were used to measure indole acetic acid (IAA), corn nucleoside hormone (ZR), abscisic acid (ABA), and photosynthesis-related parameters in leaves of *R. soongorica* seedlings at 0 (24–48 h after salt treatment), 3, 6, and 9 days. At the same time, growth indicators (salt injury index, root-to-shoot ratio), reactive oxygen species content, superoxide dismutase enzyme (SOD) activity, osmolyte content, membrane peroxidation, and leaf pigment content were measured at different salt concentrations and treatment times. Finally, principal component analysis and membership function method were used to comprehensively evaluate the salt tolerance of seedlings. The results showed that treatment with 200 mM NaCl for 3 days significantly increased SOD activity, the content of osmotic adjustment substances (proline, soluble protein), endogenous hormone content (ABA, ZR), root-to-shoot ratio, and Chla/Chlb values but decreased malondialdehyde content (MDA) in the leaves of *R. soongorica* seedlings. Leaf water content (LRWC), net photosynthetic rate (Pn), transpiration rate (Tr), water use efficiency (WUE), and IAA content in *R. soongorica* seedlings were lower than those in the control, when exposed to 400 and 500 mM NaCl solutions. Finally, the principal component analysis revealed endogenous hormone content and antioxidant enzyme activity to be useful for the comprehensive evaluation of salt tolerance in *R. soongorica* seedlings. The *R. soongorica* seedlings showed the strongest salt tolerance when exposed to 200 mM NaCl for 3 days. This study provides a theoretical foundation for gene mining and breeding of salt-tolerant species in the future.

## Introduction

Soil salinization is a prominent abiotic factor, which restricts the healthy and stable growth of global agroforestry and the sustainable development of the environment.^1^ The area covered by saline and alkali soil has reached 954 million hectares worldwide.^2^ There are about 37 million hectares of saline-alkaline land in China, accounting for 4.9% of the cultivated land.^3^ Particularly in northwest China, drought and infrequent rain, scarce vegetation, soil erosion, and desertification have resulted in the degradation of soil conditions. In addition, climate change, rise in sea levels, and release of salt from weathering rock have further increased the area of salinized land.^4^ Therefore, new varieties of salt-tolerant plants for regional cultivation are urgently needed.

Adaptation of plants to NaCl requires morphological, physiological, and biochemical changes.^5^ Different plants show different performances under salinity stress. It has been reported that relative water content and chlorophyll a and b (Chl a and b) content decreased significantly in *Cucumis melo* L. leaves with an increase in salt concentration around the plant, while an increase in proline, soluble carbohydrate content, antioxidant enzyme activity, Na^+^ concentrations in leaf and stem were noted.^6^ An increase in salinity decreases the ability of water absorption and photosynthesis in plants and inhibits their normal growth. Absorption of Na^+^ and Cl^−^ by plant roots leads to nutritional imbalance and antagonism between nutrients and salt ions.^7^ In many cases, salinity may impose osmotic stress that reduces plant productivity by impairing plant growth. However, salinity may not always harm plants. For instance, in some halophytes, low levels of salt stress can accelerate plant growth, and inhibition of plant growth occurs only at high levels of salt stress.^8,9^

Plant hormones are important chemical messengers that regulate the growth and development of plants. The balance of plant hormones is broken on exposure to salt stress, which triggers an endogenous hormone-related signal cascade, leading to downstream salt stress response in the plant.^10,11^ Studies have shown that the physiological response of Arabidopsis to salt is regulated by hormones. Under saline conditions, abscisic acid (ABA) content increases in *Arabidopsis*.^12^ With increased soil salinity, indole acetic acid (IAA) synthesis is inhibited, while ABA synthesis is stimulated, in *Helianthus tuberosus* leaves. Under conditions of salt stress, IAA/ABA values in leaves of *Helianthus tuberosus* seedlings decrease significantly.^13^ Overall, the effects of salt stress on plants depend on salt concentration, exposure time, plant genotypes, and environmental factors.^14^ When plants are subjected to salt stress, they survive via osmotic regulation, antioxidant protection, ion transport, photosynthesis, hormone regulation, and a series of physiological activities^15^ that are reflected in the plant growth index.^16^

*Reaumuria soongorica* is a super-xerophyte, salt-tolerant shrub. It is a dominant species in deserts, particularly the desert steppes in China. It has strong resistance, ecological adaptability, and plasticity for growth in the sandy conditions of deserts, and thus, plays a good role in preventing winds and fixing sand.^17^ It functions as a biological barrier against complete desertification in arid areas and creates pastures in deserts. It is very important to maintain the normal function of the desert ecosystem as it prevents soil erosion by wind, improves ecosystem restoration, and helps build ecologically secure desert societies.^18^Seedlings of *R. soongorica* are known to exhibit tolerance to a range of NaCl concentrations; NaCl may stimulate their growth and alleviate drought stress. Moderate NaCl concentrations have been reported to significantly increase photosynthetic capacity and mitigate light suppression under drought stress in *R. soongorica*. An increase in the relative water content in leaves has also been observed.^19,20^,studied the effects of salt on seed germination and early seedling growth of *R. soongorica* and found that salt may promote germ growth to some extent. As compared to germination under saline conditions, the survival of *R. soongorica* seeds under high salt conditions reflects the adaptability of this plant species to a saline environment.

Although some studies have explored the physiological mechanisms behind stress resistance in *R. soongorica*, the main focus has been on determining drought resistance, photosynthetic index, biomass, etc. under conditions of salt stress. Only a few studies have addressed changes in salt injury index and endogenous hormone content in response to salt stress of different durations. Systematic and in-depth reports using comprehensive evaluation methods are thus needed to evaluate salt tolerance in *R. soongorica*. This study uses treatments with NaCl to simulate salt stress. The effects of salinity on the growth of *R. soongorica* seedlings and their physiological response mechanism to salt stress were investigated. For this purpose, salt injury index, the production rate of $O_{2}^{-}$, superoxide dismutase enzyme (SOD) activity, root-to-shoot ratio, osmotic regulation substances, membrane peroxidation, assimilating pigments in leaves, gas exchange, and endogenous hormones were determined. Relationships between these parameters were inferred using multivariate statistical analysis methods, such as descriptive statistics, correlation analysis, principal component analysis, and membership function. An evaluation system for salt tolerance in *R. soongorica* seedlings was established. Suitable screening concentrations and indexes were determined. The mechanism of salt tolerance in seedlings of *R. soongorica* has been elaborated in detail to provide a theoretical basis for the breeding and cultivation of *R. soongorica*.

## Materials and methods

### Plant material and culture

Seeds of *R. soongorica* were provided by the Academy of Qilian Water Resource Conservation Forests Research Institute, Zhangye, Gansu, China. In the middle of April 2020, seeds of the same full size were selected for experimentation. They were first disinfected for 8 mins with 1% NaClO and rinsed six times with ultra-pure water. The seeds of *R. soongorica* were planted in a plastic nutrition bowl (diameter 4.5 cm, height 8.5 cm, bottom perforation), containing vegetative soil, quartz sand, and vermiculite (3:1:1). They were then cultivated at 25 ± 1°C and 50% humidity in the greenhouse with ventilation and natural light. When the seedlings grew to a height of ~10 cm, robust and similar *R. soongorica* seedlings were selected as the test materials. Seedlings were re-planted in a pot (23 × 13 × 14 cm) with 2.5 kg of the matrix (soil: sand, 3:1). Salt stress treatment was started 7 days after the re-plantation of seedlings.

## Experimental field

The experimental field was located at the Longqu Seed Orchard Scientific Research and Experimental station in Zhangye City, Gansu province in northwestern China. This site is in the Hexi Corridor of the depression zone at the edge of the Qilian Mountain geosyncline. Its geographical position is 100°22′E, 38°78′N at 1614 m above sea level. The study area has a temperate continental arid climate with mean annual precipitation and evaporation of 131 and 1614 mm, respectively. The substrate soil is brown desert soil (pH 8.8) with an 12.63 g/kg organic matter, 0.915 g/kg total nitrogen, 13.95 mg/kg available phosphorus, and 242.7 mg/kg available potassium.

## Experimental design

The single factor (NaCl concentration) and completely randomized block designs were used in the experiment. Seedlings of *R. soongorica* exhibiting good growth and uniform sizes were selected and divided into six groups with 60 seedlings and 3 replicates in each group (A total of 270 pots of *R. soongorica* seedlings; 4 seedlings were planted in each pot.). Salt solutions of progressively increasing concentrations (0, 100, 200, 300, 400, and 500 mM NaCl) were added to each group. To avoid osmotic shock, each target concentration was reached within 24 h by gradual addition of salt solution. NaCl solutions were prepared with deionized water, each of which was then evenly poured around the root of *R. soongorica* seedling with a syringe, while ensuring that none of the solutions were wasted. The NaCl treatment for 24–48 h was considered to be the day 0 treatment; all relevant indexes were measured after 0, 3, 6, and 9 days.

## Determination of salt injury index

Phenotypic changes in the seedling leaves were observed at 0, 3, 6, and 9 days after treatment with NaCl. The salt injury index and the survival rate of *R. soongorica* seedlings were also determined. The criteria of the classification of injury ranged from level 0 to 5 and were described as follows. Level 0: No symptoms of salt injury; Level 1: The leaf margin was yellow and withered, and other parts are intact; Level 2: Less than or equal to 10 leaves lost green coloration to become yellow and withered; Level 3: More than 10 leaves (but less than half of the plant) lost green coloration to become yellow and withered; Level 4: More than half, but not all, of the plant leaves became yellow and withered; Level 5: The whole plant lost water, wilted, and dried, leading to the death of the plant.

Salt injury index (%) *=*100 x level value x plant number) /(thehighestlevelvalue×totalplantnumber).^21^

## The ratio of root-to-shoot mass, relative water content, the production rate of $O_{2}^{-}$, and SOD activity

The total dry weight of stems, branches, and leaves above the rhizosphere was counted as the aboveground part (SDW), while the dry weight of taproots and fibrous roots was the underground part (RDW). The ratio of root to shot (R/T) = RDW/ SDW. The relative water content [LRWC) of the *R. soongorica* seedlings leaves was estimated according to the method of^22^. To measure LRWC, leaves were detached from the plant at 10 am to avoid the effects of light on water loss from the detached leaves. The LRWC was calculated using the following equation: LRWC (%] = $100\times \left(FW-DW\right)/\left(TW-DW\right)$, where FW is leaf fresh weight, DW is leaf dry weight [measured after drying the leaves at 105°C for 24 h), and TW is turgid weight, which was measured after keeping the leaves in deionized water for 24 h.

For detection of $O_{2}^{-}$ and SOD activity in *R. soongorica* leaf tissue, the procedure described by^23^ was used with some modifications. Half a gram of leaf samples was suspended in 5 mL of 50 mM Phosphate Buffered Sodium (Na-PBS, pH 7.8]; the mixture was poured into liquid nitrogen and ground, followed by centrifugation at 15000 × g at 4^°^C for 10 min. The supernatant thus obtained contained the enzyme. To measure the production rate of $O_{2}^{-}$, 0.5 mL of the enzyme solution (supernatant) was added to 0.45 mL of 50 mM Na-PBS and 0.05 mL of 10 mol hydroxylamine hydrochloride; the reaction was allowed to occur for 20 min. Another 20 minutes of reaction was allowed after adding 0.5 mL of 17 mM p-aminobenzene sulfonic acid and 0.5 mL of 7 mM α -naphthylamine. The absorbance of the n-butanol phase was measured at 530 nm after adding 1.5 mL of n-butanol. To measure the SOD activity, 20 μL of enzyme solution was added to a mixture with 3 mL of 50 mM Na-PBS contained13 mM of methionine, 75 μM nitro-blue tetrazolium (NBT), 20 μM riboflavin and 0.1 mM ethylenediaminetetraacetic acid (EDTA). The reaction mixture was illuminated for 20 min under a light source with an intensity of 4000 lux. One unit (U) of SOD [U/g FW) activity is defined as the amount of enzyme required to cause a 50% inhibition in the reduction of NBT, which is monitored at 560 nm.

## Determination of osmotic regulation substances and indexes related to membrane peroxidation

The level of osmotic adjustment substances was estimated based on the amount of proline and soluble protein content measured in plant tissue, which was determined using the method of^24^. To measure the content of proline, 0.15 g of leaf samples were mixed with 2.5 mL of 3% sulfosalicylic acid, followed by boiling in a water bath in a glass tube with a stopper for 10 min for extraction. After filtration, 2 mL of the extract (toluene as control] was mixed with 2 mL of glacial acetic acid and 2 mL of acidic ninhydrin. The solution was heated in a boiling water bath for 15 min. After cooling, 4 mL of toluene were added, oscillated, and the mixture was stratified by resting. The toluene phase was separated out to read the absorbance at 520 nm; a 1–80 mg/mL standard solution was used to draw the standard curve. The content of soluble protein was determined by the Coomassie brilliant blue method with slight modifications.^24^ Briefly, 0.15 g of leaf samples were ground into a homogenate with 5 mL of 50 mM Na-PBS (pH 7.8), centrifuged at 4000 × g at 4^°^C for 10 min. The supernatant (20 mL) was transferred to a test tube, add 3 mL Coomassie brilliant blue G-250 (Shanghai Huishi Biochemical Reagent Co., Ltd) was added to it. The absorbance was measured at 595 nm after 2 min with a UV-6100 UV–vis spectrophotometer (Metash Co. Ltd), and the protein content was determined via a standard curve.

The level of membrane peroxidation was estimated based on the amount of malondialdehyde [MDA) and electrolytic leakage measured in the plant tissue. The amount of MDA in the leaves of *R. soongorica* seedlings was estimated according to the method of^24^. For this purpose, 0.5 g of leaf sample was suspended in 2 mL of 10% chilled TCA solution and 1 g of quartz sand. The mixture was ground into a homogenate and centrifuged for 10 min at 4000 × g; 1 mL of the supernatant thus obtained was transferred to a test tube and mixed with 2 mL of 0.6% TBA solution; the test tube was then placed in boiling water for 15 min; after being cooled, it was centrifuged again. The same procedure was performed with deionized water to obtain the control. The supernatant from both was used to determine OD values at 532 nm, 600 nm, and 450 nm. The amount of MDA (μmol/g] was measured using the following equation: 6.45 × [D532-D600)-0.56 D450.

Electrolytic leakage and Na^+/^ K^+^ ratio were estimated using the method of^25^,with some modifications. To measure electrolytic leakage, 0.3 g of leaves were mixed with 20 mL of deionized water in a test tube; the contents were filtered for 10 min, stood for 20 min, and then conductivity (R1] was measured. The test tube was then put into a boiling water bath at 100^°^C for 15 min, cooled to room temperature, and conductivity (R2) was measured again. Electrolytic leakage (%) was measured using the following equation: R1/ R2 × 100%. To measure the Na^+^/ K^+^ ratio, the above and underground parts of dried *R. soongorica* were powdered and passed through a 30-mesh sieve. Half a gram of this powder was heat-digested by the H_2_SO_4_–H_2_O_2_ method.^26^ Standard calibration was carried out on an FP640 flame photometer, whereby a standard curve was drawn; quantities of Na^+^ and K^+^ ions were measured, and the Na^+^/ K^+^ ratio was calculated.

## Chlorophyll content, net photosynthesis rate, transpiration rate, stomatal conductance, and water utilization efficiency

The content of Chl a and b and carotenoids in the leaves was assessed following the methods of^27,^and Brahmi et al.^28^. For this purpose, 0.1 g of fresh leaf sample was homogenized in 20 mL of methanol, chloroform, and water (12:5:3). The procedure was done in the dark for six days. The contents of Chl a, Chl b, and carotenoids were assessed by measuring absorbance on a spectrophotometer at 665, 649, and 470 nm, respectively. Net photosynthetic rate (Pn) and transpiration rate (Tr) were measured at 10:30 ~ 11:30 in the morning by LI-6800 portable photosynthesis system (LI-COR, USA). Water use efficiency (WUE) was calculated using the formula: WUE = Pn/Tr.

## Content of abscisic acid (ABA), indole-3-acetic acid (IAA), and zeatin riboside (ZR)

The methods for extraction and purification of ABA, IAA, and ZR were adapted from the methods of Yang et al.(2013)^29^ and Zhang et al.^30^. Briefly, 1.0 g of leaf sample was added to 0.1 g of polyvinylpyrrolidone (PVPP] and then ground in liquid nitrogen with pre-cooled extract (80% (v/v) methanol containing 1 mM butylated hydroxytoluene). The contents were transferred to a 10-mL centrifuge tube, mixed well, sealed, and placed in a refrigerator at 4^°^C overnight for extraction. Centrifugation was performed at 5000 × g at 4^°^C for 30 min. The supernatant, along with 1 mL of the pre-cooled extract, was added to the remaining residue and then leached for 2 hours at 4^°^C. The suspension was centrifuged again, and supernatants were combined; the residue was discarded. The supernatants were run in a Chromosep C18 column (C18Sep-Park Cartridge, MA, USA) that was prewashed with 5 mL of 100% (v/v) and 5 mL of 80% (v/v) methanol. The filtrate was collected, and the column was then washed with 100% methanol, 100% diethyl ether, and 100% methanol, successively. The filtrate was then passed through a 0.22-mm microporous membrane and then blown dry by a nitrogen blower to remove methanol. The fraction of filtrate containing hormones was dissolved in 1 mL of phosphate-buffered saline (PBS) supplemented with 0.1% (v/v) Tween 20 and 0.1% (w/v) gelatin (pH 7.5) for enzyme-linked immunosorbent assay (ELISA) performed using an LD-SY96S, Linde, Shandong, China, and an ELISA kit (JLC-E2850, Jianglan Pure, Jiangxi, China). The antigens, antibodies against the hormones, and IgG horseradish peroxidase (HRP) were manufactured at the Institute of Biochemistry and Cell Biology (Chinese Academy of Sciences, Shanghai, China). The hormones analyzed in this study, i.e., ABA, IAA, and ZR, were quantified using ELISA according to a procedure described previously.^31^

## A comprehensive evaluation of salt tolerance

Pearson’s correlation analysis was carried out to relate 22 indexes at the seedling stage of *R. soongorica* under salt stress; the correlations among the indexes were compared. Principal component analysis was carried out on the indexes of *R. soongorica* under different salt concentration and stress duration. The dimensions of the 22 indexes were reduced, and comprehensive indexes related to salt tolerance were identified. Finally, the subordinate function method^32^ was used to evaluate the salt tolerance of seedlings in each treatment.

The weight of each index was calculated using:

$$rj=\left(\sum_{i=1}^{n}\times w_{ij}\times f_{i}\right)/\left(\sum_{i=1}^{n}\times f_{i}\right)j=1,2,3,\ldots ,\;\;n$$

where $r_{j}$ is the weight of the j index; $\;w_{ij}$ is the coefficient of the j index of the i principal component; $\;f_{i}$ is the variance contribution rate of the i principal component; and n is the number of principal components used to calculate weights.

Normalization of the weight of all indexes: $Rj=r_{j}/\left(\sum_{j=1}^{m}\times r_{j}\right)j=1,2,3,\ldots ,\;\;n$

Where $R_{j}$ is the weight of the j index after normalization; $\;r_{j}$ is the weight of the j index; m is the number of indexes.

The membership function value of each index under salt stress was calculated as:

$$U\left(X_{j}\right)=\left(X_{j}-X_{min}\right)/\left(X_{max}-X_{min}\right)j=1,2,3,\ldots ,\;\;n$$

where X_j_ is the j index value; X_min_ is the minimum value of the j index; X_max_ is the maximum value of the j index.

A value was assigned to the comprehensive evaluation of salt tolerance of each index under salt stress using the following:

$$D=\sum_{j=1}^{n}\left[u(Xj)\times R_{j}\right]\;\;\;\;\;j=1,2,3,\ldots ,\;\;n$$

## Statistical analysis

Data were processed and analyzed by Microsoft Excel 2016 and SPSS statistical software (Version 22 (IBM SPSS)). The experimental data were tested for normality and homogeneity of variances using the Shapiro–Wilk and Kolmogorov–Smirnov test with Lilliefors correction and Levene’s test. The results on the effects of treatment on proliferation met the normality assumption (α > 0.10), but they did not satisfy the assumption of homogeneity of variances (p < .20); therefore, they were analyzed using Welch’s adjusted F ratio for one-way analysis of variance (ANOVA). The Game-Howell test was used for post-hoc analysis. In the current study, figures were made by Origin 8.0 and Hiplot.

## Results

### *Effects of NaCl concentrations on salt injury index of* R. soongorica *seedlings*

The salt injury index of the leaves of *R. soongorica* seedlings showed an overall increasing trend with the prolongation of the duration of salt stress and an increase in salt concentration (Table 1). After 3 days of treatment with 100 mM NaCl, the leaves of *R. soongorica* showed slight symptoms of salt injury. When the salt stress lasted 3 days, the salt injury index of the leaves was not significantly different between 200 and 300 mM NaCl treatments; it simply increased by 11.11% and 14.58% in these two treatments, respectively, as compared to the 0 mM NaCl treatment. A sharp increase in the salt injury index was observed with an increase in salt concentration when the duration of salt stress was 6 or 9 days.

**Table 1.** Effects of NaCl concentrations on salt injury index of *R. soongorica* seedlings.

NaCl concentration (mM/L)	Salt injury index (%)			
	0 Day	3 Day	6 Day	9 Day
0	0.00 ± 0.00e	0.00 ± 0.00d	0.00 ± 0.00d	0.00 ± 0.00 f
100	0.00 ± 0.00e	0.00 ± 0.00d	4.17 ± 0.18c	12.50 ± 1.18e
200	6.25 ± 0.31d	11.11 ± 1.27c	16.67 ± 1.81b	22.22 ± 1.68d
300	8.33 ± 0.39c	14.58 ± 1.39c	30.56 ± 3.86b	40.63 ± 3.54c
400	25.00 ± 2.90b	38.54 ± 3.89b	52.08 ± 3.97a	67.50 ± 2.28b
500	47.92 ± 4.80a	62.50 ± 2.64a	70.00 ± 7.25a	83.33 ± 5.27a

Notes: Data were presented as average ± SEM (n = 12). Different lowercase letters indicate significant differences from different salt levels (p < 0.05).

## Effects of NaCl concentrations on relative water content, the production rate of $O_{2}^{-}$, SOD activity, and the ratio of root-to-shoot mass in *R. soongorica* seedlings

**Figure 1.**
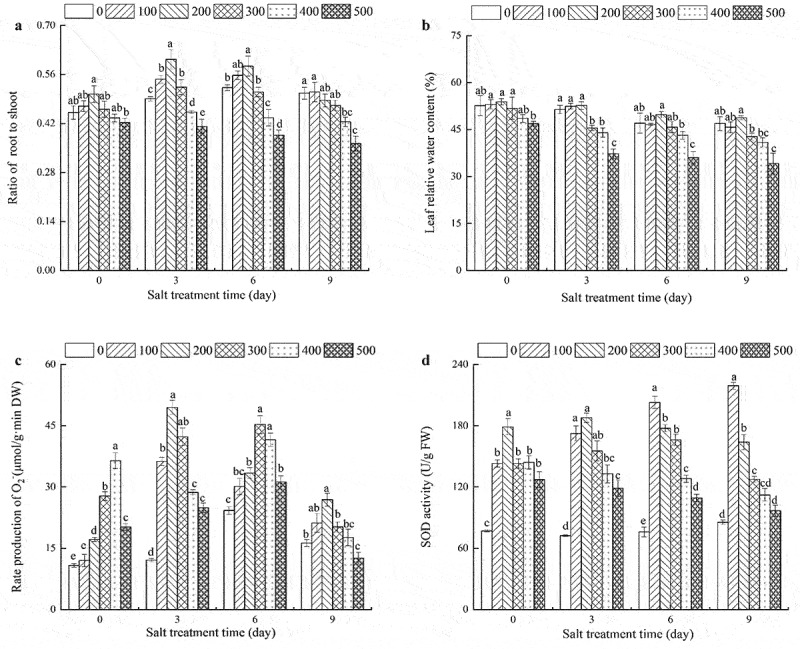
Effects of NaCl concentration on root-to-shoot ratio, relative water content, the production rate of $O_{2}^{-}$ and SOD activity of *R. soongorica* seedling leaves.

Notes: In the figure, 0,100,200,300,400,500 represent different salt treatment concentrations, the unit is expressed in mM/L. Data were presented as average ± SEM (n = 6). Different lowercase letters indicate significant differences from different salt levels (p < .05). The same as Figures 2 and 3.

With an increase in NaCl concentration, the root-to-shoot mass ratio initially increased and then decreased; the decrease was not significant (p < .05). At 0 and 9 days after NaCl stress, the ratio of root-to-shoot mass under 200 mM NaCl increased significantly; similarly, at 400 and 500 mM NaCl stress for 3 and 6 days, the root-to-shoot ratio was increased significantly. The root-to-shoot ratio reached a maximum value within 3 days of the 200 mM NaCl treatment (Figure 1a). As can be seen from Figure 1b, under the same salt stress duration, the relative water content in the *R. soongorica* seedlings in the 100 and 200 mM NaCl treatment groups increased as compared to the 0 mM treatment; the increase was not significant, and the relative water content remained stable. There was a significant difference between the 500 and 0 mM NaCl treatment groups. As compared with salt stress that lasted 3 days, the relative water content in leaves of the 100 and 200 mM NaCl treatment groups decreased by 11.08 and 5.49%, respectively, after 6 days of stress.

With the extension of salt stress duration, superoxide anion free radical production activity in *R. soongorica* leaves initially increased and then decreased (Figure 1(c)). Under 100, 200, 300, 400, and 500 mM NaCl treatments, the superoxide anion radical activity peaks (reached significant levels) occurred at 3, 3, 6, 6, and 9 h, respectively. Salt treatment increased SOD enzyme activity in *R. soongorica* leaves (Figure 1(d)). Under 100 mM NaCl treatment, the SOD enzyme activity showed a trend of gradual increase. Under 200 and 300 mM NaCl treatment, the SOD enzyme initially increased and then decreased. Under 400 and 500 mM NaCl treatments, the activity of SOD enzyme activity decreased gradually.

## Effects of NaCl concentrations on indexes related to osmotic adjustment substances and membrane peroxidation in *R. soongorica* seedlings

As compared to the treatment with no salt application, NaCl treatment increased the content of organic osmotic substances and membrane lipid peroxidation levels in leaves of *R. soongorica* (Table 2). Furthermore, the Na^+^/ K^+^ value in shoots and roots of *R. soongorica* increased as well. Within 0 days of the NaCl treatment, the indexes of *R. soongorica* seedlings under the 100 and 200 mM NaCl treatments remained largely unchanged as compared to the control. The contents of proline and MDA in leaves and Na^+^/ K^+^ value in the aboveground biomass of *R. soongorica* under the 300 mM NaCl treatment were significantly higher than in the control; these indexes increased gradually with an increase in salt concentration. After 3 days of NaCl treatment, leaf proline and MDA content, electrolytic leakage, and root Na^+^/ K^+^ values under the 200 mM NaCl treatment were significantly higher than in the control. With an increase in salt concentration, the root Na^+^/ K^+^ value initially decreased and then increased, while the other indexes only increased. There were no significant differences in leaf soluble protein, MDA, and relative electrical conductivity between 100 and 200 mM NaCl treatments on the 6^th^ day of treatment, but all indexes were significantly higher in 300–500 mM NaCl treatment than in the control. After 9 days of salt treatment, the soluble protein content in leaves initially increased and then decreased with an increase in salt concentration, while the Na^+^/ K^+^ value in roots initially increased, then decreased, and, finally, increased, while other indexes consistently increased with an increase in salt concentration.

**Table 2.** Effects of NaCl concentrations on indexes related to osmotic adjustment substance and membrane peroxidation of *R. soongorica* seedings.

Salt treatment time(day)	NaCl concentration (mM/L)	Proline content (mg/g FW)	Soluble protein content (mg/g FW)	MDA content (mmol/g FW)	Electrolytic leakage (%)	Na^+^/ K^+^ ratio in overground part	Na^+^/ K^+^ ratio in roots
0	0	364.353 ± 24.355c	0.746 ± 0.050a	5.036 ± 0.354c	38.462 ± 1.002ab	2.000 ± 0.133c	1.613 ± 0.462ab
	100	389.018 ± 43.763bc	0.714 ± 0.026a	4.968 ± 0.375c	35.065 ± 1.382bc	2.632 ± 0.215bc	1.471 ± 0.085ab
	200	402.972 ± 28.378abc	0.752 ± 0.043a	5.295 ± 0.427bc	40.154 ± 3.003a	2.500 ± 0.121bc	1.205 ± 0.140b
	300	426.400 ± 19.935ab	0.727 ± 0.016a	5.58 ± 0.153ab	33.380 ± 1.293c	2.857 ± 0.497ab	1.266 ± 0.154b
	400	438.707 ± 33.418ab	0.765 ± 0.014a	6.023 ± 0.166a	34.041 ± 2.962c	3.125 ± 0.154ab	1.563 ± 0.069ab
	500	456.593 ± 37.2990a	0.780 ± 0.025a	6.080 ± 0.082a	36.232 ± 1.795bc	3.380 ± 0.599a	1.724 ± 0.136a
3	0	487.091 ± 35.859e	0.816 ± 0.081d	6.606 ± 0.177bc	28.553 ± 1.895d	2.326 ± 0.442c	1.020 ± 0.125c
	100	626.521 ± 16.047d	0.853 ± 0.020d	6.494 ± 0.123bc	36.421 ± 2.094c	2.439 ± 0.165c	0.962 ± 0.104c
	200	808.346 ± 33.316c	0.943 ± 0.037c	5.988 ± 0.991c	38.022 ± 2.764bc	2.778 ± 0.224c	1.512 ± 0.202a
	300	1022.379 ± 49.250b	0.992 ± 0.028bc	6.212 ± 0.322bc	41.720 ± 4.808bc	3.336 ± 0.228b	1.112 ± 0.108bc
	400	1354.167 ± 41.374a	1.044 ± 0.034ab	7.016 ± 0.329ab	46.143 ± 3.516ab	3.571 ± 0.320ab	1.389 ± 0.043ab
	500	1312.557 ± 24.876a	1.104 ± 0.019a	7.682 ± 0.204a	52.050 ± 3.012a	4.000 ± 0.084a	1.515 ± 0.334a
6	0	389.018 ± 18.430d	0.868 ± 0.031d	6.941 ± 0.117de	35.162 ± 3.353c	2.222 ± 0.474d	1.429 ± 0.084c
	100	456.167 ± 20.147d	0.894 ± 0.041d	6.828 ± 0.239e	38.204 ± 3.620c	3.440 ± 0.473c	1.163 ± 0.072d
	200	643.241 ± 18.352c	0.932 ± 0.043 cd	7.480 ± 0.570 cd	41.482 ± 1.658bc	3.704 ± 0.183c	1.538 ± 0.136c
	300	957.635 ± 50.807b	0.984 ± 0.016c	7.984 ± 0.113bc	47.120 ± 2.347b	4.000 ± 0.126bc	2.083 ± 0.026b
	400	1022.379 ± 32.320b	1.056 ± 0.046b	8.516 ± 0.312b	54.322 ± 3.968a	4.545 ± 0.264b	2.381 ± 0.184a
	500	1212.913 ± 78.930a	1.141 ± 0.022a	9.196 ± 0.367a	60.561 ± 4.615a	5.556 ± 0.291a	2.500 ± 0.180a
9	0	500.608 ± 13.225e	1.182 ± 0.045e	6.787 ± 0.158e	41.650 ± 3.039c	2.703 ± 0.178 f	2.174 ± 0.182d
	100	675.743 ± 67.348d	1.182 ± 0.032d	7.024 ± 0.204e	43.742 ± 4.249c	5.000 ± 0.223e	2.500 ± 0.144 cd
	200	642.740 ± 38.286d	1.703 ± 0.025a	8.048 ± 0.314d	44.165 ± 3.920bc	4.348 ± 0.392d	2.635 ± 0.027bc
	300	976.228 ± 32.739c	1.584 ± 0.019b	8.876 ± 0.130c	52.610 ± 1.845b	5.883 ± 0.127c	2.941 ± 0.215ab
	400	1059.754 ± 29.179b	1.451 ± 0.021c	10.575 ± 0.329b	57.620 ± 2.929b	7.143 ± 0.238b	2.500 ± 0.166 cd
	500	1147.862 ± 51.916a	1.313 ± 0.029d	12.482 ± 0.836a	67.539 ± 1.586a	9.091 ± 0.164a	3.226 ± 0.349a

Notes: Data were presented as average ± SEM (n = 6). Different lowercase letters indicate significant differences from different salt levels (p < 0.05). The same as Table 3.

**Table 3.** Effects of NaCl concentrations on photosynthetic pigment content of *R. soongorica* leaves.

Salt treatment time(day)	NaCl concentration (mM/L)	Chl a (mg/g FW)	Chl b (mg/g FW)	Chlorophyll (mg/g FW)	Carotenoids (mg/g FW)	Chla/Chlb
0	0	0.585 ± 0.017a	0.331 ± 0.020a	0.916 ± 0.029a	0.188 ± 0.031a	1.766 ± 0.089a
	100	0.578 ± 0.032a	0.324 ± 0.032a	0.903 ± 0.054a	0.215 ± 0.006a	1.806 ± 0.108a
	200	0.615 ± 0.015a	0.345 ± 0.041a	0.960 ± 0.052a	0.222 ± 0.015a	1.794 ± 0.177a
	300	0.559 ± 0.067ab	0.321 ± 0.052a	0.880 ± 0.100ab	0.206 ± 0.008a	1.748 ± 0.175a
	400	0.491 ± 0.037bc	0.291 ± 0.011a	0.782 ± 0.028bc	0.197 ± 0.037a	1.692 ± 0.176a
	500	0.442 ± 0.005c	0.289 ± 0.017a	0.731 ± 0.020c	0.185 ± 0.003a	1.534 ± 0.085a
3	0	0.565 ± 0.015ab	0.353 ± 0.053ab	0.917 ± 0.051ab	0.247 ± 0.017a	1.624 ± 0.211bc
	100	0.604 ± 0.027ab	0.343 ± 0.030ab	0.948 ± 0.054ab	0.275 ± 0.022a	1.774 ± 0.055ab
	200	0.673 ± 0.034a	0.360 ± 0.005a	1.034 ± 0.030a	0.319 ± 0.037a	1.871 ± 0.102a
	300	0.584 ± 0.021ab	0.327 ± 0.024ab	0.911 ± 0.006ab	0.270 ± 0.040a	1.807 ± 0.183a
	400	0.489 ± 0.012bc	0.313 ± 0.044ab	0.803 ± 0.032bc	0.167 ± 0.020b	1.567 ± 0.188c
	500	0.414 ± 0.059c	0.280 ± 0.025b	0.693 ± 0.059c	0.147 ± 0.026b	1.486 ± 0.210c
6	0	0.522 ± 0.012a	0.350 ± 0.018a	0.872 ± 0.019a	0.242 ± 0.020ab	1.515 ± 0.083a
	100	0.493 ± 0.040a	0.345 ± 0.044a	0.838 ± 0.033ab	0.223 ± 0.010b	1.438 ± 0.267ab
	200	0.461 ± 0.045ab	0.338 ± 0.009a	0.799 ± 0.052abc	0.271 ± 0.024ab	1.367 ± 0.114bc
	300	0.401 ± 0.015bc	0.296 ± 0.012b	0.671 ± 0.025bcd	0.290 ± 0.034a	1.355 ± 0.065bc
	400	0.358 ± 0.047c	0.268 ± 0.042b	0.626 ± 0.072 cd	0.210 ± 0.028b	1.346 ± 0.230bc
	500	0.329 ± 0.055c	0.254 ± 0.028b	0.583 ± 0.037d	0.139 ± 0.012c	1.300 ± 0.418c
9	0	0.439 ± 0.045ab	0.320 ± 0.010a	0.758 ± 0.048a	0.225 ± 0.013a	1.376 ± 0.124a
	100	0.475 ± 0.033a	0.325 ± 0.023a	0.801 ± 0.012a	0.211 ± 0.014ab	1.462 ± 0.208a
	200	0.453 ± 0.006ab	0.321 ± 0.005a	0.774 ± 0.011a	0.209 ± 0.026ab	1.431 ± 0.009a
	300	0.352 ± 0.012bc	0.261 ± 0.022ab	0.613 ± 0.027b	0.172 ± 0.011bc	1.349 ± 0.119a
	400	0.322 ± 0.004c	0.239 ± 0.028b	0.540 ± 0.036b	0.156 ± 0.023 cd	1.480 ± 0.166a
	500	0.295 ± 0.008c	0.225 ± 0.013b	0.520 ± 0.013b	0.118 ± 0.012d	1.316 ± 0.094b

## Effects of NaCl concentrations on photosynthetic pigment content in *R. soongorica* leaves

There was no significant difference in the content of photosynthetic pigments between the 0 and 100 mM NaCl treatments (Table 3). With an increase in NaCl concentration, the contents of Chl a and b in the leaves of *R. soongorica* seedlings initially increased and then decreased at 0 days after salt stress. After 3 days of salt stress, the content of Chl a, chlorophyll, and the value of Chl a/Chl b were the highest in 200 mM NaCl treatments; these were 19.25%, 12.76%, and 15.21% higher than in the control, respectively. After 6 days of salt stress, carotenoid content in the 300 mM NaCl concentration was 16.55% higher than that in the 0 mM NaCl treatment; all other indexes showed a downward trend. After 9 days of salt stress, the content of photosynthetic pigments in the leaves of *R. soongorica* gradually decreased with an increase in salt concentration.

## Effects of NaCl concentrations on photosynthetic characteristics of *R. soongorica* seedlings

**Figure 2.**
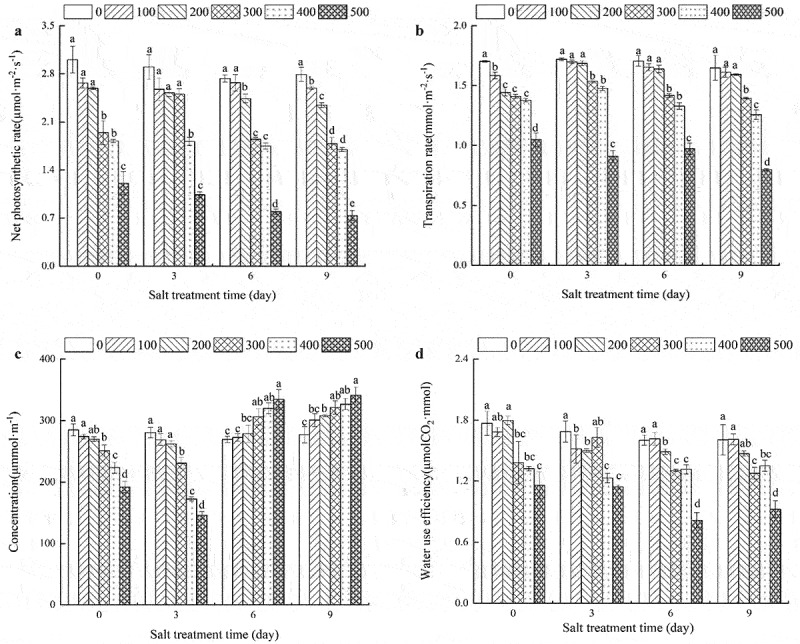
Effects of NaCl concentrations on photosynthetic characteristics of *R. soongorica* leaves.

With the same salt stress durations, the Pn and Tr of the leaves of *R. soongorica* seedlings showed a decreasing trend as the NaCl concentration increased (Figure 2a,2b). When the salt stress lasted 0 days, the Pn and Tr of the leaves treated with 300, 400, and 500 mM NaCl were significantly lower than that of the control. After 3 days of salt stress, the Pn of leaves treated with 100, 200, and 300 mM NaCl exhibited no significant difference; however, this Pn was significantly higher than that recorded for 400 and 500 mM NaCl treatments. The Tr of leaves treated with 100 and 200 mM NaCl was not significantly different as compared to that of leaves treated with 0 mM NaCl, but it was significantly higher than that of leaves from the other three treatments. After 6 or 9 days of salt stress, the Tr of leaves treated by NaCl was significantly lower than that of the control; however, the Pn of leaves from plants grown at 100 and 200 mM NaCl was not significantly different from that of the control. When the salt stress lasted 9 days, the Pn of the leaves treated with all NaCl concentrations was significantly lower than that of the control leaves.

At the same salt concentration, the CO_2int_ concentrations in *R. soongorica* leaves initially decreased slightly and then increased with time (Figure 2(c)). When the salt stress lasted 0 and 3 days, the CO_2int_ concentration showed a decreasing trend with an increase in the NaCl concentration. When the salt stress lasted 6 or 9 days, the CO_2int_ concentrations in leaves showed an increasing trend with an increase in NaCl concentrations. When the salt stress lasted 3 days, the CO_2int_ concentrations were the highest for the 0 mM NaCl treatment as compared to the 300, 400, and 500 NaCl treatments. There were no significant differences among the 100 and 200 mM NaCl treatments. When salt stress lasted 6 days, the CO_2int_ concentrations in the 300, 400, and 500 mM NaCl treatments were significantly higher than that recorded in leaves from the 0, 100, and 200 mM NaCl treatments. When the salt stress lasted 0 or 3 days, the WUE of *R. soongorica* leaves initially increased and then decreased with an increase in salt stress. The amplitude of change was significantly reduced when seedlings were treated with 400 and 500 mM NaCl concentrations as compared to the control. When the salt stress lasted 6 or 9 days, the WUE of the leaves showed a decreasing trend with an increase in salt concentrations. When the salt stress lasted 6 days, no significant difference was observed in the 100 and 0 mM NaCl treatments; in both cases, WUE was higher than in other treatments. When the salt stress lasted 9 days, the difference in WUE of plants grown at 100, 200, and 0 mM NaCl concentrations was not significant (Figure 2d).

## Effects of NaCl concentrations on endogenous hormones in *R. soongorica* seedlings

**Figure 3.**
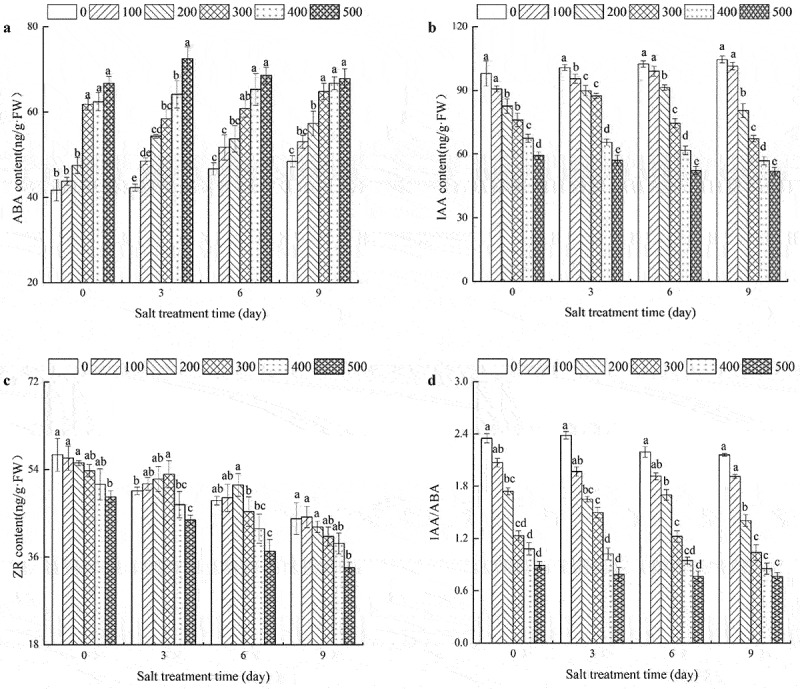
Effects of NaCl Concentrations on endogenous hormones of *R. soongorica* seedling leaves.

With an increase in salt concentration, the content of endogenous hormone, ABA, in the leaves of *R. soongorica* showed an overall upward trend (Figure 3a), while the content of IAA showed a decreasing trend (Figure 3b). When the salt stress lasted 0 days, ABA content in seedlings from the 300, 400, and 500 mM NaCl treatments was significantly higher than in seedlings from the 0, 100, and 200 mM NaCl treatments. There was no significant difference between the IAA content in the seedlings from 200 and 300 mM NaCl treatments at day 3, and no significant difference between IAA content in seedlings from 0 and 100 mM NaCl after 6 or 9 days. In the 300 mM NaCl treatment, the ABA content in leaves was the lowest, and IAA content was the highest after 3 days of exposure to salt stress. In the 500 mM NaCl treatment, with a prolongation in exposure to salt stress, ABA content initially increased and then decreased, while IAA content decreased gradually.

The content of ZR and IAA in *R. soongorica* seedling leaves showed almost a similar response to elevated salt levels, when the salt stress lasted 0 or 9 days (Figure 3c). When the salt stress lasted 3 days, the content of ZR at 100, 200, and 300 mM NaCl concentrations were slightly higher than that in the control seedlings; the content of ZR in seedlings at 300 mM NaCl concentration was 7.00% higher than that in the control seedlings. When the salt stress lasted 6 days, the content of ZR in seedlings at 100 and 200 mM NaCl concentrations was slightly higher than that in the control and was significantly higher than that in other treatments; however, ZR content was not significantly different in the two treatments. As can be seen in Figure 3d, the value of IAA/ABA decreased gradually with an increase in salt concentration. With a prolongation in exposure to salt stress, the value of IAA/ABA initially increased and then decreased with under 300 mM NaCl concentration.

## Correlation analysis among individual indexes

Note 2: SOD superoxide dismutase; OFR Superoxide anion radical; Gi Intercellular CO_2_ concentration; $R_{Na+/K+}$ Root sodium potassium ratio; Tsp Soluble protein content; $ST_{Na+/K+}$ Aboveground sodium-potassium ratio; MDA Malondialdehyde; EC Electrolytic leakage; ABA Abscisic acid; Pro Proline content; IAA Indole acetic acid; IAA/ ABA The value of indole acetic acid to abscisic acid; ZR Corn nucleoside hormone; Tr Transpiration rate; Pn Net photosynthetic rate; WUE Water use efficiency; LWC Relative water content; Chl a Chlorophyll a content; Chl b Chlorophyll b content; Chl a/b The ratio of Chlorophyll a to Chlorophyll b; Car Carotenoid contents; R/T The ratio of root to shoot. The same as Tables 4 and 5.

**Table 4.** Principal component analysis of salt tolerance evaluation of *R. soongorica* seedlings under different salt concentration and treatment time.

Variable	Weight	Principal Component1	Principal Component 2	Principal Component 3	Principal Component 4
R/T	0.049	0.414	0.375	0.620	−0.233
LWC	0.069	0.884	−0.013	0.018	0.216
OFR	0.040	0.017	−0.305	0.741	0.016
SOD	0.077	0.203	−0.067	0.842	0.177
Pro	0.045	−0.756	−0.149	0.281	−0.157
Tsp	0.006	−0.637	0.551	0.218	−0.094
MDA	0.006	−0.866	0.344	0.051	0.065
EC	0.013	−0.886	0.170	0.047	0.101
$ST_{Na+/K+}$	7.323*10^−4^	−0.887	0.274	0.038	0.160
$R_{Na+/K+}$	0.011	−0.751	0.506	−0.012	0.113
Chl a	0.049	0.872	−0.240	0.012	0.174
Chl b	0.026	0.773	0.040	−0.040	−0.447
Chl a/b	0.087	0.565	−0.376	0.062	0.599
Car	0.080	0.728	0.171	0.522	0.027
Pn	0.056	0.891	0.383	−0.065	−0.013
Tr	0.067	0.845	0.377	0.203	−0.042
Gi	0.091	−0.253	0.559	0.040	0.412
WUE	0.040	0.833	0.292	−0.221	0.005
ABA	0.061	−0.840	−0.381	0.222	−0.115
IAA	0.054	0.862	0.423	0.055	−0.097
ZR	0.022	0.846	−0.345	−0.029	0.050
IAA/ ABA	0.050	0.843	0.711	−0.150	−0.010
Eigenvalues		12.207	2.834	2.225	1.005
Contribution/%		55.486	12.880	10.114	4.566
Cumulative contribution/%		55.486	68.366	78.480	83.046

**Table 5.** The membership function value, D value and comprehensive evaluation of the leaves of *R. soongorica* seedlings under different salt concentration and treatment time.

T (day)	C _NaCl_ (mM/L)	R/T	LWC	OFR	SOD	Pro	Tsp	MDA	EC	$ST_{Na+/K+}$	$R_{Na+/K+}$	Chl a	Chl b	Chl a/b	Car	Pn	Tr	Gi	WUE	ABA	IAA	ZR	IAA / ABA	D values	RK
0	0	0.289b	0.622ab	0.012e	0.010d	0.174c	0.481ab	0.222c	0.627ab	0.057c	0.544ab	0.862ab	0.530a	0.551a	0.405a	0.896a	0.992a	0.925a	0.848a	0.122b	0.906a	0.828a	0.960a	0.579b	3
	100	0.489ab	0.654ab	0.056e	0.604b	0.339bc	0.185b	0.177c	0.352bc	0.345bc	0.408ab	0.935a	0.520a	0.676a	0.693a	0.736b	0.830b	0.846ab	0.754a	0.189b	0.749b	0.773a	0.809ab	0.632a	1
	200	0.536a	0.714a	0.239d	0.928a	0.432ab	0.537ab	0.393bc	0.764a	0.285bc	0.153b	0.796ab	0.429a	0.593a	0.773a	0.606b	0.635c	0.751bc	0.725a	0.306b	0.575c	0.691a	0.629b	0.628a	2
	300	0.608a	0.563ab	0.615b	0.604b	0.589ab	0.306ab	0.582ab	0.215c	0.447ab	0.212b	0.678b	0.360a	0.572a	0.604a	0.398c	0.589 cd	0.644c	0.414b	0.763a	0.433d	0.558ab	0.350c	0.554b	4
	400	0.337b	0.325bc	0.921a	0.615b	0.671ab	0.657ab	0.874a	0.269c	0.570ab	0.496ab	0.365c	0.150a	0.483a	0.495a	0.341c	0.545d	0.380d	0.350b	0.782a	0.246e	0.325bc	0.269c	0.461c	5
	500	0.193c	0.201c	0.345c	0.462c	0.791a	0.796a	0.912a	0.446bc	0.686a	0.650a	0.140d	0.135a	0.243a	0.371a	0.052d	0.091e	0.081e	0.166b	0.919a	0.071 f	0.117c	0.165c	0.287d	6
3	0	0.385bc	0.856a	0.012 f	0.006 f	0.033e	0.233d	0.574bc	0.148d	0.186c	0.191c	0.585bc	0.797a	0.261b	0.510b	0.904a	0.992a	0.950a	0.834a	0.043e	0.970a	0.529ab	0.887a	0.551bc	4
	100	0.473bc	0.920a	0.625c	0.833b	0.180d	0.326d	0.537bc	0.404c	0.238c	0.123c	0.903ab	0.507ab	0.849a	0.633ab	0.750b	0.967a	0.846b	0.582b	0.220d	0.858b	0.640a	0.671b	0.653b	2
	200	0.576b	0.932a	0.961a	0.961a	0.373c	0.551c	0.373c	0.457c	0.395c	0.771a	0.971a	0.532ab	0.903a	0.821a	0.612c	0.953a	0.852b	0.345c	0.387c	0.737c	0.716a	0.506c	0.741a	1
	300	0.805a	0.523b	0.778b	0.692c	0.600b	0.674bc	0.446bc	0.577bc	0.654b	0.299bc	0.655ab	0.735ab	0.376b	0.610ab	0.541 cd	0.779b	0.597c	0.398c	0.502c	0.686c	0.792a	0.426c	0.597bc	3
	400	0.257 cd	0.434b	0.432d	0.506d	0.951a	0.805ab	0.707ab	0.721ab	0.763ab	0.624ab	0.319 cd	0.424bc	0.247b	0.167c	0.426d	0.710c	0.216d	0.239c	0.666b	0.216d	0.314bc	0.178d	0.398d	5
	500	0.117d	0.051c	0.338e	0.390e	0.907a	0.955a	0.924a	0.914a	0.962a	0.771a	0.050d	0.156c	0.141b	0.077c	0.019e	0.051d	0.043e	0.031d	0.904a	0.034e	0.075c	0.056d	0.222e	6
6	0	0.570a	0.719b	0.038e	0.024 f	0.024d	0.066d	0.124de	0.111d	0.104d	0.214c	0.845a	0.763a	0.673a	0.581bc	0.995a	0.940a	0.066c	0.967a	0.069d	0.955a	0.703ab	0.924a	0.535b	4
	100	0.600a	0.694b	0.276d	0.963a	0.099d	0.147d	0.086e	0.201d	0.407c	0.042d	0.730a	0.740a	0.540a	0.850a	0.865b	0.877ab	0.102c	0.845b	0.266 cd	0.894a	0.736ab	0.753b	0.611a	1
	200	0.878a	0.888a	0.411c	0.776b	0.309c	0.266 cd	0.305 cd	0.297 cd	0.473c	0.285c	0.491b	0.576ab	0.426a	0.744ab	0.725c	0.859b	0.171c	0.664c	0.343c	0.754b	0.879a	0.621c	0.593ab	2
	300	0.806a	0.640b	0.896a	0.692c	0.661b	0.429c	0.474bc	0.463c	0.547bc	0.638b	0.374bc	0.493ab	0.386a	0.475c	0.546d	0.595c	0.477b	0.615c	0.621b	0.449c	0.580bc	0.328d	0.554ab	3
	400	0.245b	0.478c	0.744b	0.410d	0.733b	0.655b	0.653b	0.676b	0.682b	0.830a	0.205 cd	0.352ab	0.399a	0.403c	0.495e	0.489d	0.632ab	0.627c	0.793ab	0.217d	0.388c	0.157e	0.471c	5
	500	0.057b	0.034d	0.322 cd	0.271e	0.947a	0.922a	0.882a	0.859a	0.934a	0.907a	0.092d	0.279b	0.305a	0.012d	0.020 f	0.062e	0.796a	0.056d	0.922a	0.045e	0.136d	0.044e	0.297d	6
9	0	0.744a	0.859ab	0.300d	0.014 f	0.015e	0.077e	0.022e	0.087c	0.019 f	0.105d	0.865a	0.896a	0.575a	0.923a	0.951a	0.895a	0.098d	0.824a	0.066d	0.961a	0.834a	0.941a	0.579b	3
	100	0.699ab	0.794b	0.580b	0.973a	0.264d	0.268d	0.057e	0.157c	0.362d	0.306 cd	0.837a	0.862a	0.591a	0.848ab	0.757b	0.857a	0.369c	0.593b	0.267c	0.905b	0.861a	0.780b	0.640a	1
	200	0.708ab	0.960a	0.905a	0.577b	0.217d	0.951a	0.207d	0.171c	0.265e	0.389bc	0.487b	0.502b	0.490a	0.663b	0.645c	0.838a	0.449bc	0.457bc	0.453b	0.533c	0.706ab	0.451c	0.583b	2
	300	0.654ab	0.629c	0.525bc	0.315c	0.692c	0.752b	0.329c	0.452b	0.494c	0.578ab	0.366bc	0.379bc	0.464a	0.404c	0.486d	0.632b	0.601b	0.392c	0.780a	0.298d	0.559ab	0.213d	0.498c	4
	400	0.365bc	0.525c	0.377 cd	0.205d	0.811b	0.529c	0.578b	0.619b	0.682b	0.306 cd	0.257c	0.246c	0.472a	0.299c	0.404e	0.489c	0.663ab	0.387c	0.863a	0.112e	0.450b	0.093de	0.429d	5
	500	0.203c	0.154d	0.091e	0.095e	0.936a	0.297d	0.858a	0.949a	0.972a	0.753a	0.164c	0.162c	0.402a	0.051d	0.023 f	0.010d	0.830a	0.052d	0.913a	0.024e	0.070c	0.036e	0.293e	6

Notes 1: The T in the table represents salt treatment time; C _NaCl_ represents the salt treatment concentration; RK in the table represents the comprehensive ranking of salt tolerance.

Notes 2: Data were represented by an average of at three replicate measurements.

**Figure 4.**
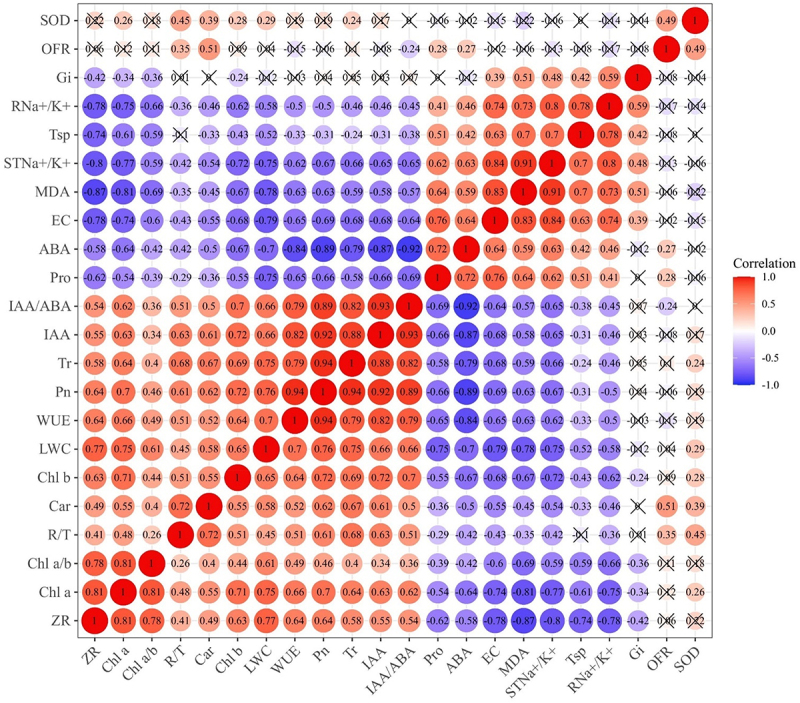
The correlation heatmap of every single index.

Correlation analysis was carried out on 22 individual indexes of *R. soongorica* seedlings under salt stress (Figure 4). Results showed that ABA, MDA, and proline contents; electrolytic leakage; and the Na^+^/K^+^ ratio in the shoot and root were negatively correlated with other indexes. The content of IAA was positively correlated with WUE, Pn, and Tr with the correlation values being 0.820, 0.920, and 0.880, respectively. Tr was positively correlated with Pn with a correlation value of 0.940. The content of ABA was negatively correlated with WUE, Pn, and IAA with the correlation values of 0.840, 0.890, and 0.870, respectively. The content of ZR was positively correlated with Chl a content and negatively correlated with MDA content. There were discrete correlations among the indexes, but the degrees of correlation were not consistent. Therefore, there was information overlap, which compromised the identification of salt tolerance indexes. Therefore, the principal component dimensionality reduction method was used to re-analyze these 22 individual indexes under different salt concentrations and treatment times to identify key players in salt tolerances.

Note 1: Pearson’s correlation identifies the relationship between two sets of data. Significance was tested at the 5% level.

## Principal component analysis for salt tolerance evaluation in *R. soongorica* seedlings under different salt concentrations and treatment times

The SPSS 22.0 software was used to perform the principal component analysis on 22 indexes under salt stress (Table 4); the Kaiser-Meyer-Olkin (KMO) value from the validity test was 0.850. Contribution rates of the first four comprehensive indicators were 55.486%, 12.880%, 10.114%, and 4.566%, while their cumulative contribution rate was 83.046%; the characteristic values were all greater than 1. The first four principal components were selected as the main factors for comprehensive evaluation of salt tolerance of *R. soongorica* based on the principle that the eigenvalue of these principal components was >1 and the contribution rate of their cumulative variance was >80%. An analysis of the characteristic vectors of the different comprehensive indexes revealed the coefficients of Pn, LWC, and Chl a to be larger in principal component 1; their values were 0.891, 0.884, and 0.872, respectively. These indexes reflect the changes in organic matter accumulated as a result of photosynthesis under salt stress. The values of coefficients of IAA/ABA, Gi, and Tsp in principal component 2 were 0.711, 0.559, and 0.551, respectively. These components reflect the changes in endogenous hormones under salt stress. The values of coefficients of SOD, OFR, and R/T in principal component 3 were 0.842, 0.741, and 0.620, respectively. They mainly reflect the changes in antioxidant enzymes in leaves of *R. soongorica* under salt stress. The values of coefficients of Chl a/b, Gi, and LWC in principal component 4 were 0.599, 0.412, and 0.216, respectively. These reflect the changes in photosynthetic pigment content in leaves under salt stress. Based on the results of principal components analysis, changes in photosynthetic parameters, endogenous hormone content, and autioxidant enzyme activities can be used as comprehensive identification indexes for the evaluation of salt tolerance at the seedling stage of *R. soongorica*.

## A comprehensive evaluation of the membership function of salt tolerance under different salt concentrations and treatment times

To comprehensively evaluate the tolerance of seedlings to salt stress, the membership function method was used. Morphological, physiological, and biochemical indexes (reactive oxygen species accumulation, autioxidant enzyme activities, ion content, osmolyte content, membrane peroxidation, photosynthetic parameters, and endogenous hormone content) were evaluated in *R. soongorica* seedlings under different salt concentrations and exposure durations. As can be seen from Table 5, according to the salt tolerance or D value (0.741) of each index, salt tolerance was found to be the highest under 200 mM NaCl treatment after a duration of 3 days. When the salt stress lasted 6 or 9 days, the highest comprehensive evaluation values were 0.611 and 0.640, respectively, under the 100 mM NaCl treatment.

## Discussion

In many parts of the world, salinity is one of the most important environmental challenges limiting plant growth.^5^ Salinity hampers photosynthesis, growth, and the formation and accumulation of endogenous hormones in plants.^13,33^ Photosynthesis is one of the most important physiological processes performed by all green plants.^34^ Salinity negatively influences the photosynthetic capacity of plants via stomatal limitation; short-term salt stress leads to osmotic stress, while long-term salt stress leads to ionic toxicity due to the imbalance of cytoplasmic nutrition[^7,35^]. In addition, the photosynthetic capacity of plants grown under saline-alkaline conditions is affected by salt concentration, exposure time, and plant species.^36–38^

The salt injury index is an important parameter to evaluate salt tolerance in a plant species.^39^ In a study on cucumber and tomato, it was found that the salt injury index increased with an increase in salt stress and duration of stress.^40,41^ Similar trends were observed here for *R. soongorica*. A mild symptom of salt injury was observed on the leaves of *R. soongorica* after 3 days of treatment with 100 mM NaCl. Data presented in Table 1 indicates an increasing salt injury index with prolongation of the duration of salt stress and an increase in salt concentration. Leaves are the primary organs for photosynthesis, where stored water can buffer rapid changes in rhizosphere salinity.^42,43^ Salinity may lead to a decrease in the relative water content but it increases the content of osmoregulatory proteins, reactive oxygen species, MDA, and proline leading to an imbalance in the inorganic ions of wheat leaves.^44^ In another study, NaCl was reported to increase electrolyte leakage and activities of antioxidant enzymes in barley seedlings.^45^ In addition, salt stress has been observed to increase the ratio of root-to-shoot in maize and promote plant growth.^46^

In this study, we observed that the antioxidant enzymes in the leaves of *R. soongorica* rapidly eliminated the harmful superoxide anion-free radical from the plant body in the 100 and 200 mM NaCl treatments. When the salt concentration was increased to 300 mM, the activities of various antioxidant enzymes increased due to the increase in osmotic substances, such as proline and soluble proteins. The damage caused by membrane lipid peroxidation to plant cells was reduced to a certain extent. When the salt concentration reached 400 or 500 mM, the water absorption capacity of roots was inhibited, resulting in severe water loss from the cells, and thus, inhibition of plant growth (Figure 1, Table 2). It was observed here that the ratio of the root-to-shoot mass of *R. soongorica* seedlings under lower concentrations (100 or 200 mM) of NaCl was the same as or even increased as compared to the ratio recorded for the control; the peak ratio was recorded on the 3^rd^ day (Figure 1), indicating that low concentrations of salt may stimulate the growth of *R. soongorica* seedlings. This could be an adaptive response to salt stress. The ability of *R. soongorica* to recover was poor at high NaCl concentrations (400 or 500 mM) as the growth of roots was inhibited (Figure 1b). On the 3^rd^ day, the recovery ability of *R. soongorica* seedlings was the strongest in the 200 mM NaCl treatment and they showed a certain salt tolerance. This is consistent with the report of^47^,on *Sesuvium portulacastrum*. When the concentration of NaCl reached 200 mM, the growth of *R. soongorica* seedlings increased noticeably and decreased beyond this concentration.

Previous studies^36,38^ have reported leaf pigments in plants to be negatively correlated with salinity. Based on our analysis and findings, leaf pigment content exhibited varying responses to different stress levels and durations. Only a slight effect of salt stress was observed on the metabolism of photosynthetic pigment in the leaves of *R. soongorica* in the 100 mM NaCl treatment. When the salt stress lasted for 3 days, the content of assimilating pigments was the highest in the leaves of seedlings grown at 200 mM NaCl (Table 3). This is a characteristic of salinity-tolerant species that can protect themselves from deterioration by increasing the stability of their chloroplast membranes.^48^ The leaf pigment content was inhibited at higher NaCl concentrations (400 or 500 mM NaCl) (Table 3) because of the salt-induced weakening of protein-pigment-lipid complex or increased chlorophyllase enzyme activity. This data is consistent with the results of^36^,and ^38^. Both stomatal and non-stomatal restrictions lead to a decrease in the photosynthetic rate of plants under stress.^49,50^ In the present study, the net photosynthetic rate, transpiration rate, and CO_2int_ concentration decreased with an increase in NaCl concentration when the salt stress lasted from 0 to 3 days. Furthermore, there was a strong linear relationship among these three indexes. The results showed that stomatal restriction was one of the main reasons for a decrease in the net photosynthetic rate of *R. soongorica* under salt stress (Figure 2). This is consistent with the results of previous studies^51^ on *Capsicum annuum* L. When salt stress lasted 6 or 9 days, the net photosynthetic and transpiration rates decreased with an increase in NaCl concentration, while the CO_2int_ concentration increased with an increase in NaCl concentration (Figure 2). These results indicated that the non-stomatal factors were the main reason for the decline of photosynthetic activity over longer durations. In such a situation, the CO_2int_ concentration could not be utilized for unknown reasons, and thus, must be further investigated.

Plant growth is closely related to the regulation of various endogenous phytohormones under abiotic stress conditions.^52,53^ Our results showed that both IAA and IAA/ABA decreased with an increase in salt stress, while salt stress promoted ABA content in *R. soongorica* seedlings (Figure 3). The content of endogenous ABA increased rapidly in a short period upon exposure to salt stress at a certain concentration (Figure 3a). This is attributable to the expression of a series of resistance genes and proteins due to increased ABA content. Our results agree with those of ^54^,and Daviere et al ^55^, who reported enhanced ABA biosynthesis in plants due to salt accumulation.^54–56^,reported salt stress to reduce IAA content in the leaves of tomatoes. Salt stress increases IAA content in leaves of cassava,^57^ which is contrary to our observations in *R. soongorica* seedlings, where IAA content initially increased and then decreased with prolongation in salt stress with 300 mM NaCl (Figure 3b). In this study, the seedlings of *R. soongorica* exhibited resistance and tolerance to salt stress. As compared to the control, the content of ZR in *R. soongorica* seedlings was slightly higher in the 100 and 200 mM NaCl treatments on the 3^rd^ and 6^th^ days (Figure 3c). The increase in ZR content promoted the growth of stem and leaves of the seedlings and enhanced stress resistance in *R. soongorica*. However, the regulation of ZR was limited in seedlings. With prolonged exposure to salt stress and an increase in salt concentration, ZR synthesis in roots decreased, resulting in a decrease in ZR content in the leaves of *R. soongorica*. These observations are not corroborated by previous studies,^56–59^ which may be attributed to inherent differences in the tolerance of different plant species.

Plant stress tolerance is a complex process regulated by multiple genes^60^; it is not reasonable to evaluate salt tolerance with a single index. The findings of ^61^,indicated relative water content and photosynthesis to be reliable indicators of salt tolerance in seedlings of upland cotton (*Gossypium hirsutum L*.).^61^ In a study with sunflower (*Helianthus annuus L*), it was found that germination index and germination vigor index were the two most reliable traits with the strongest correlation to salt tolerance.^62^ In this study, principal component analysis was used to identify photosynthetic parameters, along with endogenous hormone content and autioxidant enzyme activities in leaves. These may be used as screening indexes for salt tolerance in *R. soongorica* seedlings (Table 4). Based on the principal component analysis, in combination with the membership function value and the corresponding linear weighting, salt tolerance value was determined by comprehensive evaluation. It was found that *R. soongorica* seedlings showed the strongest tolerance to salt at 200 mM NaCl concentration for a duration of 3 days (Table 5). These results are consistent with the growth indexes identified under different salt concentrations and treatment times.

## Conclusion

This study presents the different growth and physiological response strategies, as well as morphological and physiological characteristics, of *R. soongorica* seedlings under different salt concentrations and exposure times. Salt stress can damage cell membrane by lipid peroxidation and change the content of osmotic adjustment substances, antioxidant enzyme activity, chloroplast ultrastructure in the palisade tissue, gas exchange parameters, and endogenous hormone content in leaves. Under short-term (3 days) mild salt stress (200 mM NaCl), the salt injury index and membrane lipid peroxidation levels in leaves of *R. soongorica* seedlings were low. However, SOD activity; content of ABA, ZR, and osmotic regulation substances; and the net photosynthetic rate were high. *R. soongorica* seedlings eliminated harmful superoxide anion free radicals from the plant body and reduced oxidative damage by controlling the content of osmotic regulators and antioxidant enzyme activity in ion-stable leaves. This, in turn, promoted the growth and development of plants, allowing them to adapt to salt stress. High salt concentrations of 400 and 500 mM NaCl caused significant changes in endogenous hormones, increased the content of osmotic adjustment substances, reduced antioxidant enzyme activity, lowered photosynthetic pigment synthesis, and destroyed the photosynthetic structure of leaf cells, resulting in an imbalance in intracellular environmental homeostasis, cell metabolism disorders, and severely inhibited the growth of seedlings. In combination with a comprehensive evaluation system for salt tolerance, it was found that the seedlings showed the strongest salt tolerance at 200 mM NaCl on the 3^rd^ day of exposure. This study lays a theoretical foundation for breeding salt-tolerant species and gene mining in the future.
